# Breast papillary lesions: an analysis of 70 cases

**DOI:** 10.3332/ecancer.2014.461

**Published:** 2014-09-02

**Authors:** Dahiana Pulgar Boin, Jaime Jans Baez, Militza Petric Guajardo, David Oddo Benavides, Maria Elena Navarro Ortega, Dravna Razmilic Valdés, Mauricio Camus Apphun

**Affiliations:** 1 Department of Oncology and Maxillofacial Surgery, College of Medicine, Pontifical Catholic University of Chile, Marcoleta 352, Santiago 8330033, Chile; 2 Department of Pathologic Anatomy, College of Medicine, Pontifical Catholic University of Chile, Marcoleta 352, Santiago 8330033, Chile; 3 Department of Radiology, College of Medicine, Pontifical Catholic University of Chile, Marcoleta 352, Santiago 8330033, Chile

**Keywords:** breast, papillary lesions, breast carcinoma

## Abstract

**Introduction:**

Papillary breast lesions are rare and constitute less than 10% of benign breast lesions and less than 1% of breast carcinomas.

**Objective:**

To analyse the clinical presentation, preoperative evaluation, and surgical and anatomopathological characteristics of the patients operated on for papillary breast lesions.

**Material and Methods:**

Retrospective descriptive and analytical study. We analysed the database of patients with definitive histopathological diagnosis of papillary breast lesions operated on at our institution from January 2004 to May 2013.

**Results:**

During the period described, 70 patients with histopathological diagnosis of papillary breast lesions were operated upon. The median age was 50 years (19–86 years). Thirty-seven patients (52.8%) were symptomatic at diagnosis. Preoperative ultrasound was reported to be altered in all patients. A mammography showed pathologic findings in only 50% of cases. All patients underwent partial mastectomy, after needle localisation under ultrasound, if the lesion was not palpable on physical examination. The final pathological diagnosis was: benign papillary lesion in 55 patients (78.6%) and malignant in 15 patients (21.4%). Adjuvant treatment was performed in all malignant cases. Median follow-up was 46 months (3–115 months).

**Conclusions:**

Patients with papillary breast lesions presented with symptoms in half of all cases. There was a high frequency of malignancy (21.4%), therefore surgical resection was recommended for papillary breast lesions.

## Introduction

Breast papillary lesions are characterised by growth inside the milk ducts, and they represent a heterogeneous pathology. They are rare and constitute less than 10% of benign breast lesions and less than 1% of malignant breast neoplasms [1–3]. Benign forms include intraductal papilloma, classified as central, peripheral, or atypical. Malignant papillary lesions may apply to non-invasive forms (intraductal or intracystic papillary carcinoma and micropapillary intraductal carcinoma) or invasive (invasive papillary and micropapillary carcinoma) [3].

Breast papillary lesions are usually detected by imaging or clinically by the presence of a palpable breast mass or unilateral spontaneous nipple discharge [4]. Radiological findings suggestive of papillary breast lesions are rare and of low specificity for the diagnosis of malignancy, since they cannot categorically distinguish benign lesions from those that are potentially malignant [5, 6].

The treatment is controversial. There is a consensus that papillomas with atypical pathological features justify surgical excision, because they present with a high rate of coexisting carcinomas [7]. However, there is no agreement on the optimal treatment of papillomas without atypia [8–12].

Our objective was to analyse the clinical presentation, the preoperative evaluation, the surgical management, and the anatomopathological characteristics from patients operated on in our facility for breast papillary lesions.

## Materials and methods

**Design:** Retrospective analytical–descriptive study.

**Patients:** An analysis was made from the database of patients with definite histophatological diagnosis of breast papillary lesions that were operated on in our facility from January 2004 until May 2013.

**Sources:** Prospective recording of clinical history and biopsies from the Clinical Hospital’s Department of Pathologic Anatomy from the Pontifical Catholic University of Chile.

**Variables:** The following were considered: age, clinical presentation form, imaging studies, histopathology, type of surgery performed, adjuvant treatment, recurrence.

**Statistical analysis:** For the statistical analysis the Statistical Package of Social Sciences (SPSS) 20.0 program was used. A descriptive and analytical statistical study was performed. To compare tests, parametric and non-parametric hypothesetic trials were run according to the normality determination. Significant confidence level of 95% with a p-value <0.05 was considered statistically. These are considered to be normal values.

## Results

During the period described 70 patients with definitive histological diagnosis of breast papillary lesions underwent treatment. The median age was 49.5 years old (19 to 86 years old).

### Clinical Presentation

The presentation form was only with radiologic findings 33 patients (47.2%), and 37 patients (52.8%) were symptomatic. Of these symptomatic, 27 patients (73%) were checked because of symptoms of nipple discharge, and ten patients (27%) because of the presence of palpable nodules at the time of the physical breast exam. Table 1 summarises the patients’ clinical characteristics.

### Radiological Findings

**Figure 1.**
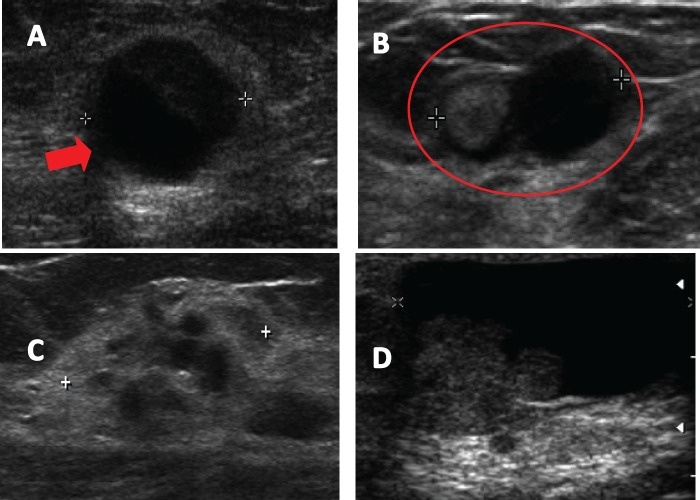
Ultrasound images described in breast papillary lesions. A. Complex solid cystic nodule. B. Lactiferous duct dilated hypoechoic content. C. Poorly defined hypoechoic nodule. D. Cystic lesion with mural pedunculated mass.

In all patients bilateral breast ultrasound and mammography was performed. Preoperative breast ultrasound was altered in all patients, reporting an intracystic nodule in 25 patients (35.6%) (Figure 1A), dilated lactiferous duct with hypoechoic content in 22 patients (31.4%) (Figure 1B), ill-defined hypoechoic solid nodule in 14 cases (20%) (Figure 1C), and cystic lesion with mural pedunculated mass in nine patients (12.9%) (Figure 1D), with an average lesion size of 9.8 ± 4.2 mm.

The mammography revealed the presence of nodules reported as Breast Imaging Report and Database System 4 (BI-RADS 4) in 31 patients (44.3%), benign findings (BI-RADS 2) in 23 patients (32.9%), regular analysis (BI-RADS 1) in 12 patients (17.1%), and in four cases density asymmetry presence, shown as BI-RADS 4 (5.7%).

**Figure 2.**
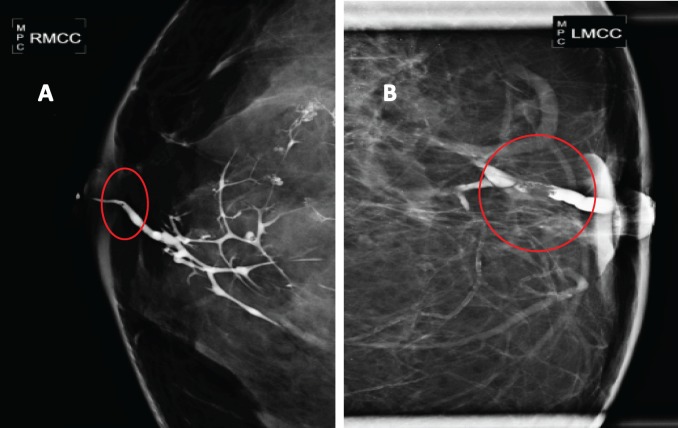
Galactography suggestive of breast papillary lesions. A. Galactography with ductal tree display from the affected duct, showing a small retroareolar filling defect (in a circle). B. Galactography with filling defect (in a circle) in a magnified mammographic plate.

In seven patients (10%) who showed up for the diagnosis with nipple discharge, a galactography was performed, which revealed in all cases the presence of a filling defect in the affected milk duct (Figure 2)

### Pathologic Anatomy

A histopathological study was conducted preoperatively with a core biopsy in 19 patients (27.1%), which showed papilloma without atypia in 12 patients (63.1%), papilloma with atypia in three patients (15.8%), and the presence of papillary carcinoma in four cases (21.1%). In 14 of the 19 patients biopsied with core needle (73.7%), the biopsied image was described in the ultrasound as an ill-defined solid nodule.

**Figure 3.**
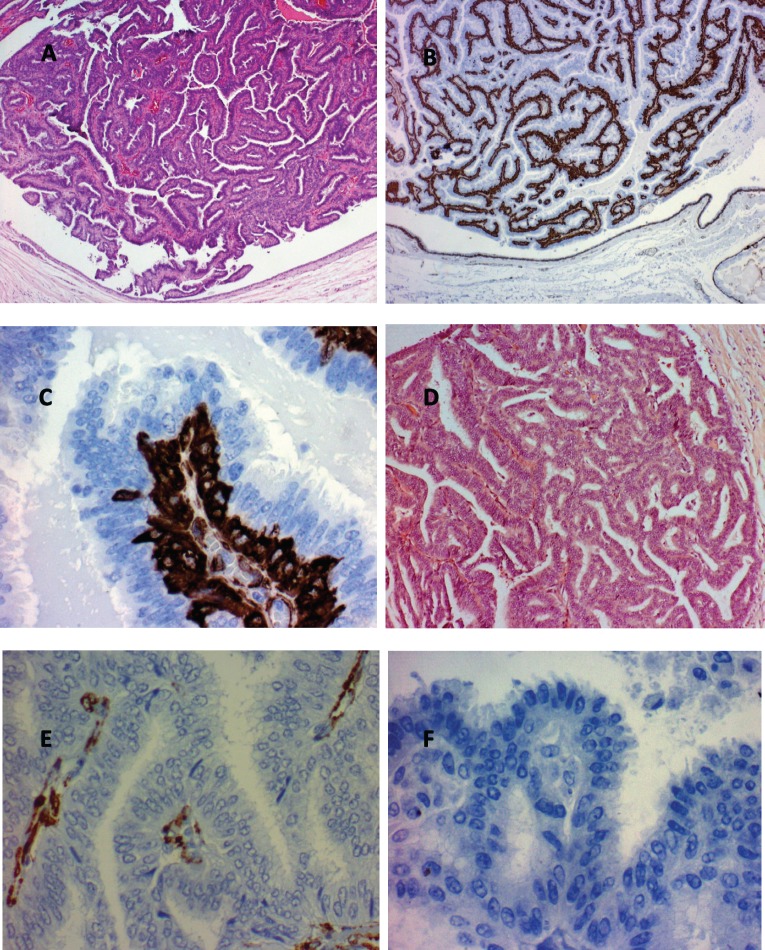
Histological findings characteristic of papillary breast lesions. A. Intracystic papilloma (hematoxylin and eosin x80). Note the marked buds with relatively broad fibrovascular cores. B. Intracystic papilloma (immunohistochemistry for actin x80). Note the presence of continuous basal strata of myoepithelial cells in all buds. C. Intracystic papilloma (immunohistochemistry for actin x80). Note the presence of abundant immunoreactive actin myoepithelial cells in the basal strata of a papilla. D. Intracystic papillary carcinoma well differentiated (hematoxylin and eosin x80). Note buds with very narrow fine and indistinct fibrovascular cores. E. Intracystic papillary carcinoma well differentiated (Immunohistochemistry for actin x400). Note the non-immunoreactive to actin epithelial cells, the absence of myoepithelial cells and the presence of some myofibroblastic cells in the papillae fibrovascular fine cores. F. Well-differentiated intracystic papillary carcinoma (immunohistochemistry for p63 (myoepithelial marker) x400). Note the absence of myoepithelial cells in the buds.

The final pathologic–anatomical diagnosis was benign papillary lesion in 55 patients (78.6%), and malignant in 15 patients (21.4%), which corresponded to papillary carcinoma *in situ* in 13 cases (86.6%), invasive papillary carcinoma in one case (6.7%) and in one patient papillary carcinoma *in situ* associated with invasive lobular carcinoma (6.7%). Table 2 summarises the pathologic–anatomical findings of final biopsy and Figure 3 proves the histological findings characteristic of benign and malignant breast papillary lesions.

When comparing the pathologic–anatomical core biopsy result with that of the surgical tissue, an underestimation of malignant lesions was observed. The core biopsy identified only four out of eight carcinomas described in the final biopsy.

Thirty-two patients (58.2%) with benign papillary lesions on the surgical tissue’s histology showed symptoms at diagnosis. Of the 15 patients with papillary breast carcinoma, only five patients (33.3%) had first appearance of symptoms (*p* = 0.08). No significant differences in radiological findings were found between patients with benign histology and those with malignant histology (Table 3).

### Surgical Treatment

**Figure 4.**
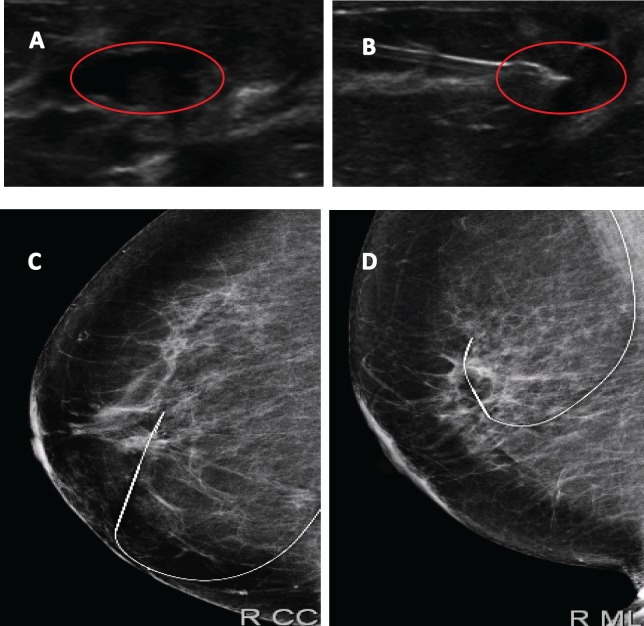
Image of papillary lesion and preoperative marking. A. Dilated milk duct with hypoechoic content. B. Marking on ultrasound lesion to be dried up. C and D Metal guide in mammographic projections LCC and LML.

In all patients, partial mastectomy, upon previous marking with the ultrasound, was performed if the lesion was not palpable on physical examination (Figure 4). In cases of non-palpable lesions, an ultrasound of the surgical tissue was performed. In three patients (4.3%) the surgical tissue biopsy reported positive for papillary carcinoma edges, so an extension of surgical margins was performed in deferred form. In two patients (2.9%) with the presence of invasive carcinoma a sentinel lymph node biopsy was performed in a second surgical operation.

An adjuvant treatment was performed in all malignant cases. Radiation therapy to the breast was given and then hormone therapy if there was a presence of oestrogen-positive receptors and in the absence of any contraindication. One patient with invasive carcinoma received chemotherapy with doxorubicin associated with cyclophosphamide (AC) for four cycles; it was well tolerated. The follow-up median was 46 months (3–115 months), without recurrences to date.

## Discussion

Breast papillary lesions are characterised histopathologically by the presence of fibrovascular stroma lined by epithelial and myoepithelial cells. They range from benign lesions such as ductal papillomas to the presence of invasive breast papillary carcinoma.

During the period described, 70 patients with definitive histological diagnosis of breast papillary lesions underwent treatment. Thirty-seven patients (52.8%) had symptoms at diagnosis, with the most common symptom being unilateral secretion (serous or serosanguineous) through the nipple, which is consistent with the literature [3, 4].

All patients in our cohort had abnormal preoperative imaging (ultrasound and/or mammography). Thirty-five patients (50%) had abnormal mammogram, with the most frequently reported finding being the presence of a nodular contour image (BI-RADS 4), consistent with the published literature. The benign papillary lesions are commonly described in the literature as a well-defined, round, or oval unique mass causing no distortion of breast parenchyma, typically in the subareolar region with presence of calcifications or microcalcifications only in 25% of the cases [13, 14]. Papillary carcinomas, on the contrary, usually show up in mammography as an ill-defined mass with stromal distortion. The presence of lobed margin, spiculation, and pleomorphic microcalcifications have also been described in this type of lesion [13, 15, 16].

The best test for detecting image papillary lesions is the breast ultrasound. In our study, 100% of patients showed an altered ultrasound. Various studies report an increased sensitivity of ultrasound to detect breast papillary lesions compared to mammography [13–15]. Breast papillary lesions’ sonographic appearance is variable, however, they have been grouped into two categories: intraductal mass, often associated with dilated duct, or as a solid mass, which may present a cystic component, but is not associated with dilated milk ducts [13]. In our patients, preoperative breast ultrasound mostly reported presence of intracystic nodule in 25 patients (35.6%) and dilated milk duct with hypoechoic content in 22 patients (31.4%). No significant differences in radiological findings between patients with benign histology and those with malignant histology were found.

Other imaging studies have also been used for the diagnosis and characterisation of patients with breast papillary lesions who show clinical nipple discharge. Galactography, which involves injecting a special dye cannulating the affected milk duct and then subsequent imaging with mammography plates, allows for the display of benign papillary lesions as filling defects associated with ductal ectasia, with the possibility of the milk duct’s amputation being present secondary to an obstruction that prevents the passage of the special dye. Papillary carcinomas in the galactography may also show pleomorphic calcifications, nodules, ductal distortion, or cystic formations communicated with the duct in question [17–19]. On the other hand, the ductoscopy directly displays the ductal tree through endoscopy exploration through optical fibre, providing information about the extent of the disease; while allowing biopsies and more selective surgeries [20, 21]. Kamali *et al* report a sensitivity for the diagnosis of breast papillary lesions from galactography and ductoscopy as 81.4 % and 86.6 % respectively [22].

In our study, only 19 patients (27.1%) underwent core biopsy under ultrasound, with 14 cases being the presence of an ill-defined solid nodule described in preoperative images, in which there was no suspicion of a papillary lesion. When comparing the pathologic–anatomical core biopsy result with the surgical tissue, it was observed that the biopsy only identified 50% of malignant lesions. Several studies show an underestimation of biopsied breast papillary lesions with core needle in ultrasound close to 25% of the cases. Rizzo *et al* reported on 101 cases of intraductal papillomas without atypia, an underestimation of malignant lesions in 24.5% of the cases [23]. In a series of Jaffer *et al* published in 2009 from 104 patients with core biopsy diagnosis of breast papillomas without atypia, 16.4% showed atypia or malignancy on operatory biopsy [24]. Wen *et al* in meta-analysis recently published included a total of 34 studies with 2,236 non-malignant breast papillary lesions diagnosed by core biopsy, reported an underestimation of malignancy in 15.7% of lesions [CI 95%: 12.8 to 18.5%], with the factors associated with a higher underestimation and the presence of atypical papillary lesions (*p* < 0.001), positive mammographic findings (*p* = 0.022), and the article was published before 2005 (*p* < 0.05) [25]. A recent study concluded that larger tissue samples (12 G needle, 7 samples take or> 96 mm3) significantly improve the predictive value of percutaneous biopsy for the diagnosis of benign papillary lesions, presenting a negative predictive value of atypia or malignancy of 100% [26].

The decision to perform percutaneous biopsy for an ultrasound lesion with suspected papillary lesion has advantages and disadvantages.

### Advantages of performing percutaneous biopsy

1. Avoiding unnecessary surgery of lesions showing dilated milk duct with hypoechoic content because it may be confused with a papillary lesion with thick-discharge ductal ecstasia that would not require surgery.
2. Knowing the preoperative diagnosis of a given lesion. If there is confirmation of carcinoma, better margins and freezing biopsy confirmation can be obtained. If invasive carcinoma are confirmed, a sentinel lymph node biopsy can be performed during the same surgery.

### Disadvantages of performing percutaneous biopsy

1. Underestimation of malignant lesions, as previously mentioned. This can be reduced with the use of thicker needles (Mammotome, Suros), but have a much higher cost to the patient.
2. Complete disappearance of the lesion. This would be an advantage for small benign lesions, but a problem for malignant lesions requiring further surgery. For this reason, it would be necessary to leave a marking clip on all dried lesions, which also raises the cost.

The frequency of malignancy in our study was 21.4%. In a nationwide study of 29 patients with breast papillary lesions [27], an association with papillary carcinoma is 13.8% as reported, concluding that this pathology as indication for surgery remains questionable.

## Conclusions

Half of the patients operated on for breast papillary lesions in our study were shown as being asymptomatic. We prefer to perform surgical resection of lesions with radiological suspicion of papillary lesion, mainly due to the underestimation of risk in malignancy and confirmed papillary carcinoma in over 20% of our study. There are cases in which prior percutaneous biopsy can be helpful, but it is suggested that its use be discussed with the patient. We did not find clinical or radiological predictors that help predict the presence of malignancy in the surgical sample. Given the high frequency of malignancy (21.4%) found, we believe that resection of these lesions is recommended.

## Conflicts of interest

The authors declare that they have no conflicts of interest.

## Figures and Tables

**Table 1.** Characteristics of patients with definitive histopathological diagnosis of breast papillary lesion (*n* = 70).

Variable	No. of patients
**Age**	
• ≤50 years	39
• >50 years	31
**Diagnosis presence of symptoms**	
• Asymptomatic	33
• Symptomatic	37
**Diagnosis symptoms**	
• Nipple discharge	27
• Palpable nodule	10

**Table 2.** Anatomopathological findings of surgical biopsy (*n* = 70).

Variable	No. of patients
**Benign**	
• Papilloma without atypia	52
• Papilloma with atypia	3
**Malignant**	
• Papillary carcinoma *in situ*	13
• Invasive papillary carcinoma	1
• Papillary Carcinoma *in situ* + invasive lobular carcinoma	1

**Table 3.** Comparison of radiological characteristics of patients with anatomopathological findings, benign and malignant (*n* = 70).

Variable	Benign (*n* = 55)	Malignant (*n* = 15)	*P*
**Ultrasound Findings**			
• Intracystic nodule (+/-)	20/35	5/10	0.82
• Dilated duct with content (+/-)	20/35	2/13	0.08
• Solid nodule (+/-)	9/46	5/10	0,14
• Cystic lesion with mural pedunculated mass (+/-)	6/49	3/12	0.315
• Size of lesion (mm) ± SD	9.2 ± 5.9	11.8 ± 8.7	0.196
**Ultrasound Findings**			
• Altered Mammography (B4)	26/29	9/6	0,38
